# CDK5 positively regulates Notch1 signaling in pancreatic cancer cells by phosphorylation

**DOI:** 10.1002/cam4.3916

**Published:** 2021-05-07

**Authors:** Qiaoyun Chu, Liyong Wang, Jie Zhang, Wei Wang, Youxin Wang

**Affiliations:** ^1^ Department of Biochemistry and Molecular Biology School of Basic Medical Sciences Capital Medical University Beijing China; ^2^ Core Facilities for Molecular Biology Capital Medical University Beijing China; ^3^ Beijing Key Laboratory of Clinical Epidemiology School of Public Health Capital Medical University Beijing China; ^4^ Centre for Precision Health School of Medical and Health Sciences Edith Cowan University Perth Australia

**Keywords:** CDK5, cell proliferation, Notch1, pancreatic ductal adenocarcinoma, phosphorylation

## Abstract

The marked overexpression of cyclin‐dependent kinase 5 (CDK5) or Notch1 receptor, which plays critical roles in pancreatic ductal adenocarcinoma (PDAC) development, has been detected in numerous PDAC cell lines and tissues. Although, a previous study has demonstrated that CDK5 inhibition disrupts Notch1 functions in human umbilical vein endothelial cells, the mechanism underlying Notch1 activation regulated by CDK5 remains unclear. Herein, we identified a physical interaction between CDK5 and Notch1 in PDAC cells, with the Notch1 peptide phosphorylated by CDK5/p25 kinase. CDK5 blockade resulted in the profound inhibition of Notch signaling. Accordingly, CDK5 inhibition sensitized PDAC cell proliferation and migration following Notch inhibition. In conclusion, CDK5 positively regulates Notch1 function via phosphorylation, which in turn promotes cell proliferation and migration. The combinational inhibition of CDK5 and Notch signaling may be an effective strategy in the treatment of PDAC.

## Graphical abstract

CDK5 and Notch1 interact physically in PDAC cells. CDK5 blockade resulted in the profound inhibition of Notch signaling. CDK5 inhibition sensitized cells proliferation and migration following Notch inhibition.

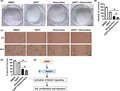

## 1 INTRODUCTION

Pancreatic ductal adenocarcinoma (PDAC) is a disease of nearly complete lethality presenting a median survival time of 6 months, and is predicted to become the second leading cause of cancer‐related deaths before 2030.^1^ This may be explained by the vast majority of patients presenting with advanced stages of PDAC at diagnosis, and PDAC cells possess a high metastatic potential and are often resistant to available cytotoxic drugs.^2^ Thus, there is an urgent need to identify aberrant signaling pathways that present suitable substrates for targeted therapies.

Notch signaling is an evolutionarily conserved cell fate determination pathway that has great relevance in multiple aspects of cancer biology.^3^ It promotes the initiation and progression of PDAC and could be a valuable target in PDAC therapy.^4^, ^5^ Furthermore, it is a juxtacrine cell‐cell communication pathway that positively or negatively affects cellular proliferation, survival, differentiation, and apoptosis in a context‐dependent manner. Notch1 is present at the cell surface as a heterodimeric molecule (p120/p200), comprising an extracellular domain (p200) involved in ligand binding and a cytoplasmic domain (p120) involved in signal transduction. The precursor Notch1 protein (p300) is cleaved into extracellular and cytoplasmic domains in the trans‐Golgi network by furin. Ligand binding triggers a cascade of prototypic cleavage events and finally releases the Notch intracellular domain (NICD). Then, the NICD is translocated into the nucleus and binds to the DNA‐binding protein CBF1, leading to the transcriptional activation of multiple effector genes such as those encoding members of the HES/HEY family.^6^ Although, the molecular components of Notch signaling have been well defined, and signaling from the cell surface to the genome is simple and linear, the mechanisms regulating Notch activity need to be comprehensively elucidated.^6^

Cyclin‐dependent kinase 5 (CDK5) is also known to contribute to PDAC formation and progression.^7^ This proline‐directed serine/threonine kinase is activated following association with either one of two noncyclin activator proteins, p35 and p39. The cleavage of p35 and p39 to p25 and p29, respectively, yields activators with greater stability and increased CDK5 kinase activation when compared with that of observed with the full‐length forms of p35 and p39.^7^ CDK5 is mainly activated by p35 or its truncated product, p25, and the active CDK5/p25 kinase is present in Golgi membranes in neurons.^8^, ^9^ Although, the role of CDK5 in the central system is well characterized, it has been recently associated with the development and progression of multiple cancers, including brain, breast, lung, colon, and pancreatic cancer.^7^, ^10^ Accumulating evidence has implicated CDK5 as an important determinant of malignant progression, invasion, and metastasis in PDAC.^11^, ^12^ CDK5 is widely active, and p35 and p39 are significantly overexpressed in PDAC.^13^ Thus, both CDK5 and Notch1 are important proteins that affect PDAC cell growth, invasion, and metastasis. In the current study, we investigated the crosstalk between CDK5 and Notch1 signaling in PDAC cells.

## 2 MATERIALS AND METHODS

### 2.1 Cell culture and chemical compounds

Human BxPC‐3 and HPAC PDAC cells were obtained from National Infrastructure of Cell Line Resource (Beijing, China) and were grown in DMEM (Dulbecco's Modified Eagle's medium) supplemented with 10% fetal bovine serum (Cat#900‐108, Gemini), 100 units/mL penicillin, and 100 mg/mL streptomycin (Cat#15140122, HyClone) at 37°C in humidified air with 5% CO_2_. siRNAs and plasmids were transfected using Lipofectamine 2000 (Cat#11668030, Invitrogen) according to the manufacturer's instructions. Roscovitine (Cat#R7772, Sigma) was used to inhibit CDK5 kinase activity. The γ‐secretase inhibitor DAPT (Cat#D5942, Sigma) was used to prevent Notch cleavage and nuclear entry. The inhibitors were diluted in dimethyl sulfoxide (DMSO), which was also used as a control.

### 2.2 Co‐immunoprecipitation and immunoblotting assay

Cells were washed with cold PBS, collected by scraping, and lysed in lysis buffer (50 mM Tris‐HCl [pH 7.5], 150 mM NaCl, 0.3% Nonidet P‐40, and 2 mM EDTA) containing protease inhibitor cocktail (Cat#HY‐K0011, MCE). Cells were lysed on ice for 40 min, followed by centrifugation at 12000 × g for 40 min at 4°C, and the supernatant was removed. For immunoprecipitaion, 1 mg of protein was incubated with 2 μg of specific antibodies at 4°C overnight. Subsequently, 30 μL protein A/G agarose (Cat#sc‐2003, Santa Cruz Biotech) was added, and samples were incubated at 4°C for 2 h. Beads were then washed five times using the lysis buffer. Between washes, the beads were collected by centrifugation at 1000 × g for 3 min at 4°C. The precipitated proteins were eluted from the beads by resuspending the beads in 2 × protein loading buffer and boiling for 10 min. Immunoblotting was then performed. For routine immunoblotting, cells were washed twice with cold PBS, then lysed in RIPA buffer. Equal amounts of protein were loaded onto SDS–PAGE and transferred onto polyvinydene fluoride (PVDF) blotting membranes. The membranes were blocked with 5% fat‐free milk then probed with indicated antibodies at 4°C. The primary antibodies were as follows: anti‐Notch1 (Cat#sc‐32756, Santa Cruz Biotech), anti‐CDK5 (Cat#LS‐B353, LifeSpan BioSciences), anti‐p35 (Cat#41304, Signalway), anti‐HA (Cat#sc‐7392, Santa Cruz Biotech), and anti‐β‐actin (Cat#66009, Proteintech Group). Members were then washed and incubated with anti‐rabbit or anti‐mouse horseradish peroxidase‐conjugated antibodies (Cat#SA00001‐1, SA00001‐2, Proteintech Group) for 1 h at room temperature. Signals were detected using a chemiluminescence Western Blot scanner (ChampGel 7000, SAGECREATION). Protein levels were normalized to β‐actin levels and quantified using ImageJ software. Densitometry analyses of the blots for Figure 3 were in Figure S2.

### 2.3 RNA extraction and expression analysis

Total RNA from HPAC and BxPC‐3 cells that had undergone indicated treatment was isolated using Trizol (Cat#15596018, Invitrogen). For reverse transcription and polymerase chain reaction (RT‐PCR), first‐strand cDNA was synthesized form 1 μg of total RNA using SuperScript Kit (Cat#18091050, Invitrogen) according to the supplier's manual. Quantitative RT‐PCR was performed using a SYBR^®^ Green Realtime PCR Master Mix (Cat#QPK‐201, Toyobo) and an Mx3000P instrument (Stratagene Laboratories). The primer sequences for qPCR are listed in Table 1.

**TABLE 1:** Sequences of the primers for qPCR

Gene	Forward primers	Reverse primers
*p35*	5’‐CCAGAACAACATCACGCACC −3’	5’‐GGGTGAGGGGCTTTCTTGAC −3’
*CDK5*	5’‐GCACAAGAACATCGTCAGGC −3’	5’‐TTGGCCCCAAAGAGGACATC −3’
*Notch1*	5’‐GCAGTTGTGCTCCTGAAGAA −3’	5’‐CGGGCGGCCAGAAAC −3’
*Hes1*	5’‐TGATTTTGGATGCTCTGAAGAAAGATA−3’	5’‐GCTGCAGGTTCCGGAGGT−3’
*C‐Met*	5’‐GTAAGTGCCCGAAGTGTA−3’	5’‐TTTCTTGCCATCATTGTC−3’
*β‐actin*	5’‐GTGACGTGGACATCCGCAAAGAC−3’	5’‐TCAAGAAAGGGTGTAACGCAACTAA−3’

### 2.4 DNA constructs, site‐directed mutagenesis, and siRNA‐mediated CDK5 knockdown

Wild‐type CDK5 was cloned into the pCDNA3.1 vector. Point mutations were established using the Q5 Site‐Directed Mutagenesis Kit (Cat#E0554, New England Biolabs) with primer pairs matching target regions. The following primers were used for mutagenesis: D144 N forward primer, 5’‐GAAATTGGCTAATTTTGGCCTGG‐3’ and reverse primer, 5’‐AGCTCCCCATTCCTGTTTATTAG‐3’; and K33 T forward primer, 5’‐GTGGCTCTGACACGGGTGAGG‐3’ and reverse primer, 5’‐GATCTCATGAGTCTCCCGG‐3’.

siRNA specific for CDK5 was synthesized by Shanghai GenePharma Co., Ltd. (Shanghai, China). The CDK5 siRNA sequence was 5’‐GGGAGAUCUGCCUACUCAATT‐3’. The negative control siRNA (NcRNA) sequence was 5’‐UUCUCCGAACGUGUCACGUTT‐3’.

### 2.5 Immunofluorescence

For cells immunostaining, cells were rinsed twice in PBS and fixed for 15 min in 4% paraformaldehyde/PBS at room temperature. After rinsing, permeabilization was achieved in 0.4% Triton X‐100/PBS for 20 min. Indicated antibodies incubation were performed in PBS overnight at 4°C, following blocking in 10% serum/PBS for 10 min. After being rinsed in PBS, cells were incubated with Alexa Fluor 488‐conjugated anti‐mouse IgG (Cat#R37120, Invitrogen) and Alexa Fluor 594‐conjugated anti‐rabbit IgG (Cat#R37117, Invitrogen) for 1 h at room temperature. Cells were DAPI‐treated to stain nuclei and mounted with anti‐fade medium. Images were captured using a confocal laser‐scanning microscope (LSM880, Carl Zeiss).

Human pancreatic cancer tissue microarrays were purchased from Xi'an Alena Biotechnology Co., Ltd. of China. Following dewaxing, rehydration and antigen retrieval, the samples were stained with Notch1 and CDK5 antibodies at 4°C overnight. The samples were then incubated with Alexa Fluor 488‐conjugated anti‐rabbit IgG (Cat#R37118, Invitrogen) and Alexa Fluor 594‐conjugated anti‐mouse IgG (Cat#R37121, Invitrogen) for 1 h at room temperature. Nuclei were conterstained with DAPI. The stained tissues were visualized using a confocal laser‐scanning microscope (LSM880, Carl Zeiss).

### 2.6 In vitro kinase assays and mass spectrometry

Assays were performed using 100 µM Notch1 peptide (LifeTein, Beijing, China) and 7.8 nM CDK5/p25 (Cat#14‐516‐M, Sigma) in 5 mM MOPS (pH 7.2), 5 mM MgCl_2_, 1 mM EGTA, 0.4 mM EDTA, 0.25 mM DTT, 50 ng/μL BAS, and 100 µM ATP. The reactions were performed for 30 min at 32.6°C. Following the kinase assays, the Notch1 peptide was subjected to TiO_2_ affinity chromatography according to the manufacturer's manual (GL Sciences, Eindhoven, Netherlands). Liquid chromatography‐tandem mass spectrometry (LC‐MS/MS) was performed using a Q Exactive mass spectrometer (Thermo Fisher Scientific, USA). The peptide‐based enzyme activity assay is a highly sensitive and convenient method.^14^

### 2.7 Expression and survival analysis using GEPIA web tool

The online database Gene Expression Profiling Interactive Analysis (GEPIA, http://gepia.cancer‐pku.cn/) was used to analyze Notch1, CDK5, p35, and p39 RNA sequencing expression data based on The Cancer Genome Altas (TCGA) and the Genotype‐Tissue Expression (GTEx) projects.^15^ The overall survival analysis was performed using Kaplan–Meier in the GEPIA dataset.

### 2.8 Cell metabolism viability, proliferation, and migration assay

BxPC‐3 and HPAC cells were inoculated in 96‐well culture plates, and cell viability was evaluated by reduction of MTT tetrazolium salt [3‐(4, 5‐dimethylthiazol‐2‐yl)‐2, 5‐diphenyltetrazolium bromide] (0.5 mg/ml) by viable cells to form purple formazan product after 2 h incubation. Experiments were performed in triplicate on indicated conditions, and repeated at least three times.

For a colony assay, 1000 cells were plated in six‐well plates to assess the effect of CDK5 and/or Notch inhibitors on colony formation after 10 days of incubation at 37°C in 5% CO_2_ incubator.

For a migration assay, HPAC cells were seeded in a six‐well plate (2 × 10^5^ cells/well) and incubated for 24 h. A scratch was made using a 1‐mL pipette tip, followed by treatment of cells with indicated inhibitors. Images were captured at 0 and 48 h using an inverted microscope. ImageJ software was used to calculate the area of the scratch. Then, the percentage of wound closure was calculated and compared with that of the control.

### 2.9 Statistical analysis

Data are presented as the mean ±standard deviation. Comparisons between control and target data sets were performed using independent sample t‐tests, with P‐values less than or equal to 0.05 indicating statistical significance.

## 3 RESULTS

### 3.1 Colocalization of CDK5 with Notch1 in vivo

**FIGURE 1:**
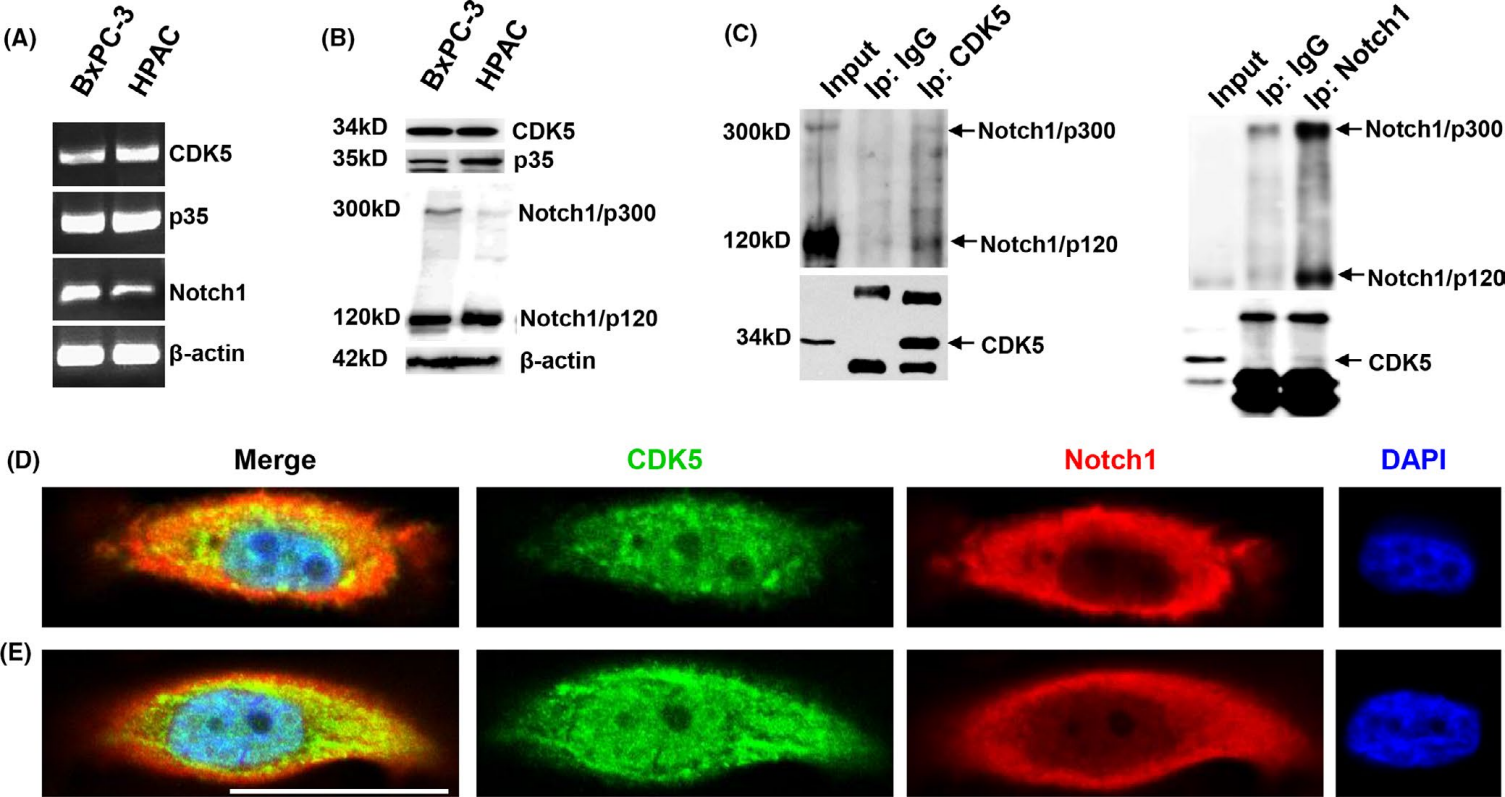
CDK5 physically associates with Notch1 in pancreatic cancer cells. The mRNA (A) and protein levels (B) of CDK5, p35, and Notch1 were examined in BxPC‐3 and HPAC cells. Cell lysates were immunoprecipitated with anti‐CDK5 (C) or anti‐Notch1 antibodies (D), and immunoblotting was performed with anti‐Notch1 and anti‐CDK5 antibodies. Normal mouse or rabbit IgGs were used as negative controls. Intracellular interactions of CDK5 and Notch1 in BxPC‐3 (E) and HPAC (F) cells were visualized with a confocal microscope. The scale bar is 25 μM. CDK5, cyclin‐dependent kinase 5

To determine the physiological relevance, we examined the interaction of CDK5 and Notch1 in vivo using co‐immunoprecipitation. CDK5, p35, and Notch1 were expressed in BxPC‐3 and HPAC cells (Figure 1A and B). We observed that Notch1 copurified with CDK5 but not with the IgG control, and CDK5 copurified with Notch1, demonstrating their specific binding and indicating a physical interaction between CDK5 and Notch1 (Figure 1C and D). To further determine whether CDK5 and Notch1 colocalize in vivo, immunostaining was performed. Staining with CDK5‐specific antibodies revealed that endogenous CDK5 was distributed through both the nucleus and cytoplasm of cells. Colocalization of Notch1 and CDK5 in the cytoplasm of BxPC‐3 and HPAC cells was observed by yellow fluorescence when images of Cy3‐stained anti‐Notch1 and FITC‐stained anti‐CDK5 immunocomplexes were merged (Figure 1E and F).

### 3.2 Phosphorylation of Notch1 peptide by CDK5 in vitro

**FIGURE 2:**
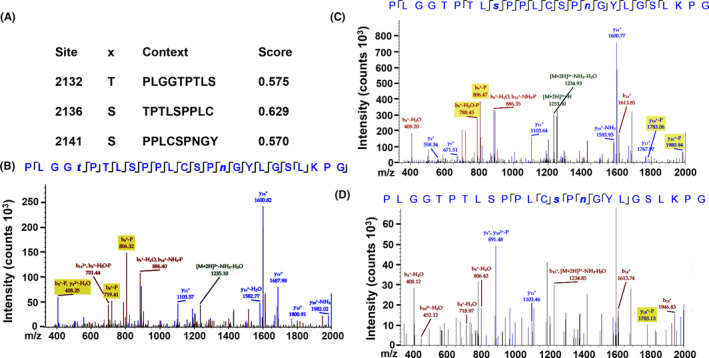
Human Notch1 contains putative CDK5 phosphorylation sites and could be phosphorylated by CDK5. (A) The human Notch1 protein sequence (NP_060087) was analyzed using the Motif Scan program. The scores of three potential CDK5 phosphorylation sites in Notch1 2128–2152 residues are shown. (B, C, D) The phosphorylated Notch1 peptides were detected by liquid chromatography‐mass spectrometry (LC‐MS). The phosphorylated S and T residues are indicated by lowercase letters. CDK5, cyclin‐dependent kinase 5

To determine the mechanism through which CDK5 regulates Notch1 activation, we explored the potential phosphorylation of Notch1 by CDK5. We analyzed Notch1 for the presence of phosphorylation motifs using the NetPhos 3.1 Server (http://www.cbs.dtu.dk/services/NetPhos/), which predicts protein phosphorylation sites and potential kinase activity. This analysis revealed the presence of 29 CDK5 phosphorylation sites in human Notch1 with a score greater than 0.5. To determine whether CDK5 affects the phosphorylation of Notch1, we determined whether CDK5 could phosphorylate a Notch1 peptide (Notch1 2128–2152, NP_060087.3) containing three potential CDK5 phosphorylation sites (Figure 2A). An in vitro kinase reaction demonstrated that T2132, S2136, and S2141 were CDK5‐phosphorylated sites in the Notch1 peptide (Figure 2B, C, and D). In the control experiment, the peak of Notch1 peptide was observed at 2424.2 kD (Figure S1A) without CDK5/p25 kinase, which shifted to 2504.8 kD with CDK5/p25 kinase (Figure S1B), indicating the specific kinase activity of CDK5 on the Notch1 peptide.

### 3.3 Inhibition of CDK5 activity impairs the Notch1 pathway

**FIGURE 3:**
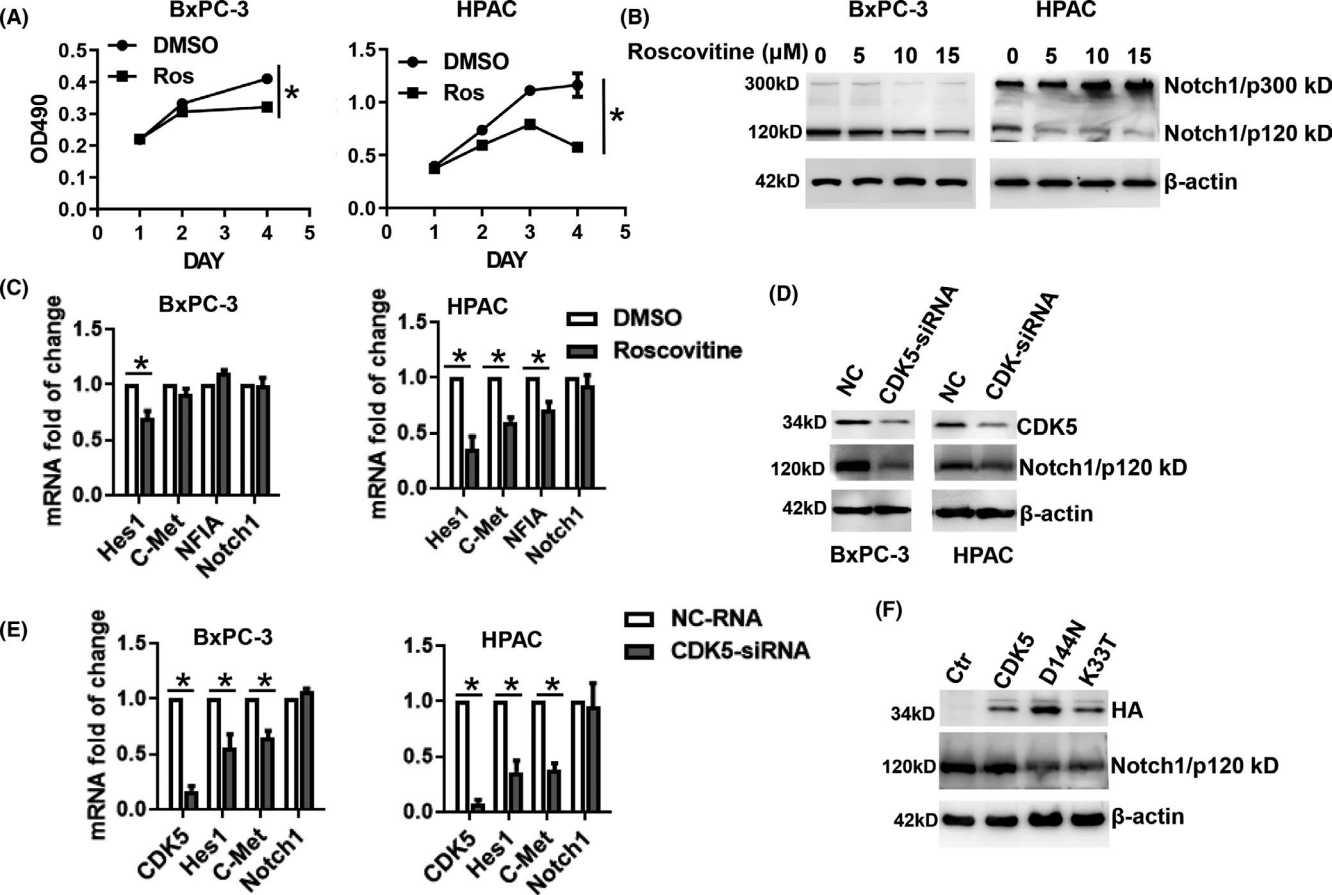
Inhibition of CDK5 attenuates Notch1 signaling. (A) Cells were treated with 10 μM roscovitine. Cell viability was detected using the MTT assay. Experiments at all‐time points were assayed in triplicate. (B) Cells were treated with indicated roscovitine concentration for 1 h, and lysates were immunoblotted with Notch1 antibody to detect the full‐length (Notch1/p300 kD) and 120 kD cleavage products of Notch1 (Notch1/p120 kD). (C) The mRNA levels of Notch1 signaling pathway effectors, Hes1, C‐Met, NFIA, and Notch1, were detected in BxPC3 and HPAC cells. Cells were treated with 10 μM roscovitine for 6 h. (D) Cells were transfected with CDK5‐siRNA or negative control‐RNA (NC) for 48 h. Protein levels of CDK5, Notch1 (Notch1/p120 kD), and β‐actin were detected. (E) The mRNA levels of CDK5, Notch1, Hes1, and C‐Met were detected using qRT‐PCR analysis. (F) HPAC cells were transiently transfected with CDK5 or CDK5‐D144 N or CDK5‐K33 T or pCMV vector as control (Ctr). HA‐tag antibody was used to detect CDK5 or CDK5 mutants. Protein levels of Notch1 (Notch1/p120 kD) and β‐actin were detected. CDK5, cyclin‐dependent kinase 5; qRT‐PCR, real‐time quantitative reverse transcription‐polymerase chain reaction

To investigate whether CDK5 regulates Notch1 cleavage and activation, CDK5 kinase activity was inhibited using roscovitine, CDK5‐specific siRNA, or dominant‐negative CDK5 (dnCDK5), respectively. The blockade of CDK5 by roscovitine markedly inhibited the viability of both BxPC‐3 and HPAC cells, as assessed by the MTT assay (Figure 3A). After pretreatment with the indicated concentration of roscovitine for 1 h, levels of the 120 kD cleavage product of Notch1 decreased in both cell lines, and full‐length Notch1 only accumulated in HPAC cells (Figure 3B). Furthermore, mRNA levels of the downstream Notch target genes, Hes1, c‐Met, and NFIA, were reduced in HPAC cells treated with roscovitine for 6 h when compared with their levels in control cells, while only the Hes1 mRNA level was decreased in BxPC3 cells. Roscovitine did not affect the Notch1 mRNA level in either cell line (Figure 3C). Although, CDK5 is the primary target of roscovitine, it also inhibits other CDKs, such as CDK1, CDK2, CDK7, and CDK9. To determine the specific effect of CDK5 blockade on Notch1, we used an RNA interference (RNAi) strategy to antagonize CDK5 function. Similarly, the Notch1 level was profoundly decreased following RNAi knockdown of CDK5 in BxPC‐3 and HPAC cells (Figure 3D). We further confirmed that mRNA levels of the Notch1 target genes Hes1 and c‐Met were downregulated in CDK5 knockdown cells; however, the mRNA level of Notch1 was not altered in either cell line (Figure 3E).

To identify whether the regulation of Notch signaling was related to the kinase activity of CDK5, we treated HPAC cells with the enforced expression of two dominant‐negative CDK5 (dnCDK5) proteins with an HA tag. The dnCDK5 constructs were D144 N and K33 T kinase‐dead mutants that abolish the activation of endogenous CDK5 by p35 when overexpressed.^16^ Consistent with results from the RNAi strategy, HPAC cells expressing dnCDK5 constructs presented significantly reduced levels of the 120 kD cleavage product of Notch1, indicating that CDK5 kinase activity decreases Notch1 activation (Figure 3F). Furthermore, the striking downregulation of the active form of Notch1 (NICD) was observed in HPAC cells with knockdown of CDK5, and could be restored by exogenous expression CDK5 (Figure 3). Collectively, these results from multiple independent experiments confirmed that Notch1 activation is profoundly disrupted in CDK5‐inhibited cells.

### 3.4 Blockade of CDK5 and Notch synergistically inhibits growth and migration

**FIGURE 4:**
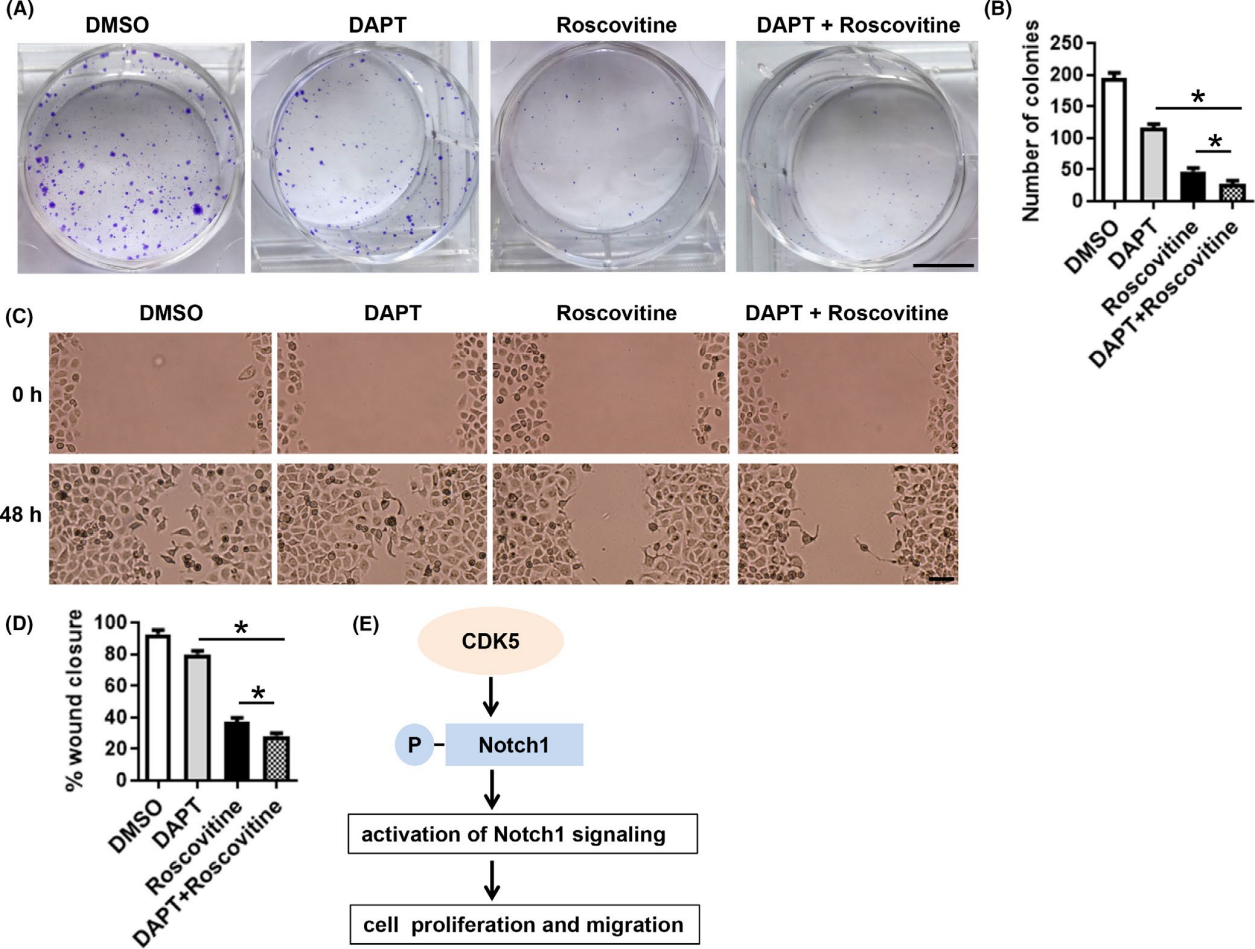
Roscovitine and DAPT synergistically inhibit proliferation and migration. HPAC cells were treated with 10 μM roscovitine and/or 2 μM DAPT. (A) Representative images showing the ability of HPAC cells to form colonies. Scale bar, 5 mm. (n = 3) (B) The histogram shows the number of colonies formed. (C) Representative microscopic images presenting the effect of roscovitine and DAPT on the migration of HPAC cells. Scale bar, 100 μM. (n = 3) (D) The semi‐quantitative image analysis of migration is also presented. (E) The proposed model presenting the function and mechanism of Notch1 regulated by CDK5. CDK5 positively regulates Notch1 signaling by phosphorylation, which in turn promotes cell proliferation and migration. *p ≤ 0.05. CDK5, cyclin‐dependent kinase 5

To determine the combined effect of CDK5 and Notch1 signaling on pancreatic cancer cells, we measured the effect of blocking CDK5 and/or Notch signaling on cell proliferation and migration. DAPT is a γ‐secretase inhibitor that inhibits the Notch signaling pathway. As several references have demonstrated that pharmacologic inhibition of CDK5 or Notch signaling had the same effect as knockdown of CDK5^12^, ^13^, ^17^ or Notch1^18^ with siRNA. Both pharmacologic and genetic inhibition mitigated growth in pancreatic cancer cells. Here, we used the inhibitors to explore the combined effect of CDK5 and Notch1 signaling on pancreatic cancer cells growth. Both DAPT and roscovitine significantly inhibited HPAC cell growth (Figure 4A) and migration (Figure 4C) when compared with that of observed in control (DMSO‐treated) cells. The combined use of DAPT and roscovitine inhibited cell growth and migration to a greater extent than treatment with either agent alone (Figure 4A and C). These results indicated that CDK5 inhibition sensitized HPAC cells to more effectively inhibit cell proliferation and migration via Notch inhibition.

### 3.5 CDK5 signaling is associated with Notch signaling in PDAC

To verify the relevance of CDK5 signaling and Notch signaling in PDAC pathogenesis, we examined the expression and interaction between CDK5 and Notch1 in clinical PDAC specimens.

**FIGURE 5:**
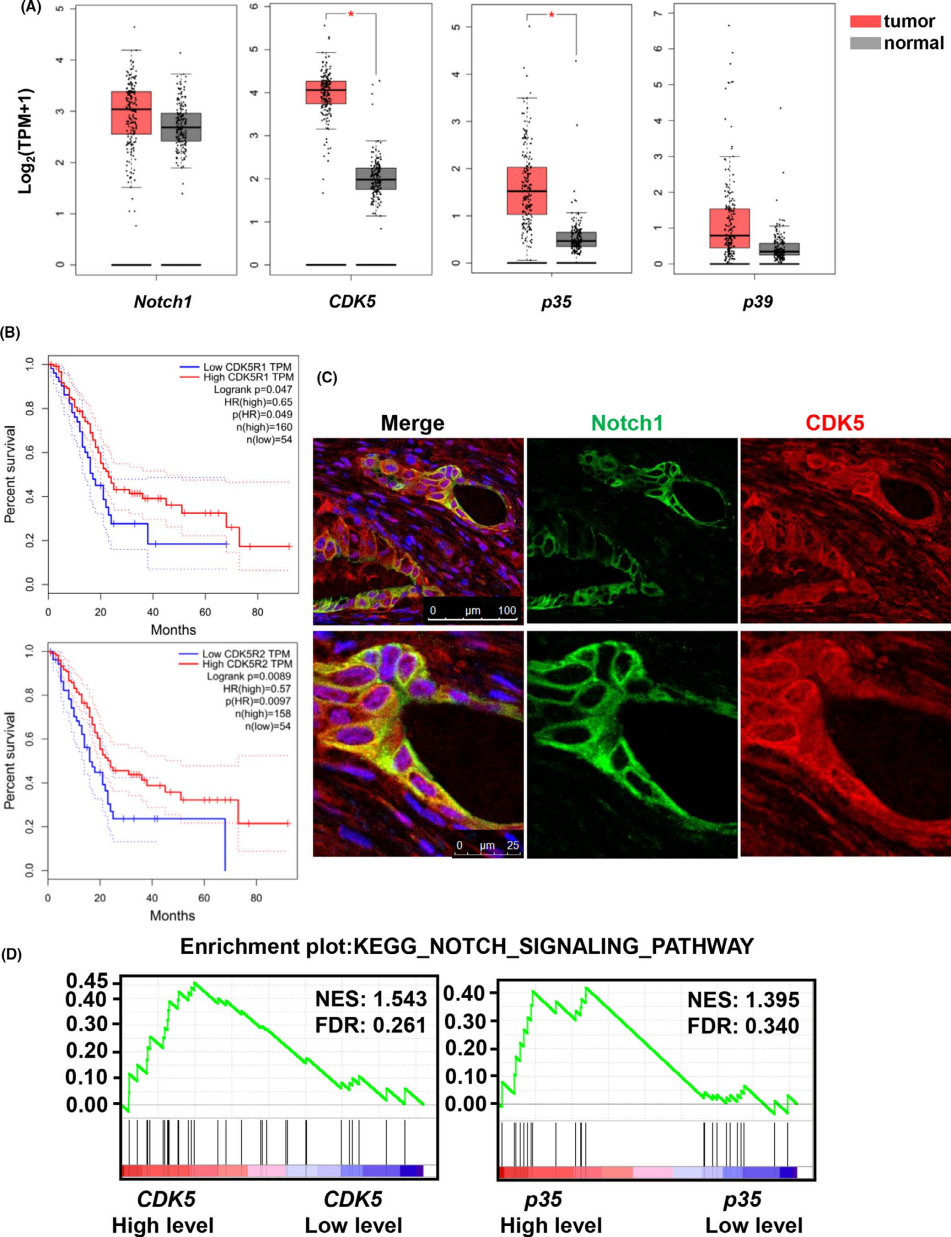
High CDK5 and p35 expression positively correlate with Notch signaling. (A) Analysis of differential expression of Notch1, CDK5, p35, and p39 in normal and PDAC patients of using GEPIA. Tumor: n = 179; Normal: n = 171. (B) The survival curves of p35 and p39 were analyzed using GEPIA. (C) Representative double immunofluorescence staining of Notch1 (green) and CDK5 (red) in PDAC. (n = 3) (D) The PDAC samples from TCGA database were ranked from low to high CDK5 or p35 expression groups, with quartile as boundary, the first 25% of the samples were the low expression group, and the last 75% were the high expression group. Enrichment plots of gene expression signatures for the Notch pathway activation was analyzed by GSEA according to CDK5 or p35 mRNA expression levels. CDK5, cyclin‐dependent kinase 5; NES, normalized enrichment score; FDR, false discovery rate

Based on the GEPIA database, we observed that *Notch1*, *CDK5*, *p35*, and *p39* were overexpressed in pancreatic adenocarcinoma (PAAD) samples (Figure 5A). The higher Notch1 and p39 expressions correlated with poor overall survival (Figure 5B). Consistent with their colocalization in HPAC and BxPC3 cells, CDK5 and Notch1 colocalized in PDAC specimens (Figure 5C). To investigate the potential relationship between Notch and CDK5 signaling pathways in patients with PDAC, Gene Set Enrichment Analysis (GSEA) using the TCGA pancreatic cancer data set was performed to assess Notch/CDK5 signaling. The GSEA outcomes revealed that gene signatures of Notch signaling activation were enriched in patients with high CDK5 expression and high p35 expression (Figure 5D), suggesting a positive correlation between Notch and CDK5 signaling.

## 4 DISCUSSION

In the present study, we unraveled the physical association between CDK5 and Notch1 and revealed their colocalization in the cytoplasm. During intracellular processing, full‐length precursor Notch1 is transported to the trans‐Golgi apparatus and then cleaved into two linked peptides.^6^ As active CDK5/p25 kinase is also located in Golgi membranes,^8^, ^9^ the two proteins might colocalize in the Golgi network. The phosphorylation of Notch1 by CDK5 could regulate the cleavage of Notch1 by furin, as well as the maturation of the Notch1 receptor.

The inhibition of CDK5 by roscovitine significantly reduced the 120 kD cleavage product of Notch1 in both BxPC‐3 and HPAC cells, which resulted in the significant accumulation of full‐length Notch1 in HPAC cells (Figure 3A). The different regulatory mechanisms of Notch activation and heterogeneity in the transcriptional activation of target genes in Notch‐ON cells might account for this observation.^5^ Consistent with a previous study, our results suggest that CDK5 has a positive effect on Notch1‐dependent signaling.^19^ In the previous report, the authors revealed that CDK5 inhibition disrupted NICD generation and Notch function.^19^ Although, BxPC‐3 and HPAC cells almost have the same expression level of CDK5 protein, the full‐length of Notch1/p300 is a little higher in BxPC‐3 (Figure 1B). In the following experiments, CDK5 inhibitor, CDK5‐siRNA and CDK5 dominant‐negative plasmids were used to explore the specific role of CDK5 in Notch1 signaling regulation, and the results showed that Notch1 activation is profoundly disrupted in CDK5‐inhibited cells (Figure 3).

The inhibition of CDK5 decreased Notch1 cleavage but not Notch1 expression, further downregulating Notch1 signaling (Figure 3). This indicates that Notch1 is regulated by CDK5 at the post‐transcriptional level. As CDK5 is a proline‐directed serine/threonine kinase, Notch1 may be regulated by CDK5 via phosphorylation. Notch is regulated by various post‐translational modifications, including phosphorylation, during multiple steps in the signaling pathway. Phosphorylation occurs at multiple sites within the NICD and regulates Notch signaling at different levels, such as the maturation and localization of Notch1 in the cell membrane,^20^ translocation of the NICD into the nucleus,^21^ the formation of a transcript complex and its binding to the promoter of the target genes,^22^ and the stability of the NICD.^23^ Three serine/threonine kinases that interact with Notch1 have been identified: GSK‐3β, CDK8, and CK2.^21^, ^24^, ^25^ In this study, we demonstrated that T2132, S2136, and S2141 are CDK5 phosphorylation sites in the Notch1 peptide. CDK5 phosphorylates proteins at two consensus sequences: (S/T)PX(K/H/R) and PX(S/T)P (where X is any amino acid).^26^ Thus, T2132, S2136, and S2141 are potential candidate sites for CDK5 phosphorylation in Notch1 in vivo. The future study needs to examine the identified Notch1 phosphorylation site with full‐length Notch1 and the effect of these sites mutations on Notch1 signaling regulated by CDK5.

High expressions of CDK5, p35, or p39 and hyper‐activation of CDK5 signaling have been reported in several cancers, including breast, ovarian, and colorectal cancer.^27^ Inhibition of CDK5 reportedly impairs tumorsphere formation and reduces tumor establishment.^28^ Based on the GEPIA database, we observed that expression levels of Notch1, CDK5, p35, and p39 are higher in PDAC tumor tissues than in normal tissues. High expression of Notch1 and p39 correlated with poor prognosis and shorter patient survival in PDAC (Figure 5A and 5B). Collectively, these observations indicate that CDK5 and Notch1 act as oncogenes in PDAC. Although, Notch1 is a target for PDAC therapy, and the downregulation of Notch contributes to the inhibition, apoptosis, and metastasis of PDAC, clinical experience with Notch pathway inhibitors remains relatively limited.^29^ Currently, several classes of Notch pathway inhibitors, including γ‐secretase inhibitors (GSIs) and specific receptors or antibodies, are in clinical development.^29^ Despite the toxicity and limited therapeutic efficacy in clinical and preclinical studies, GSIs have revealed some clinical benefits; for example, the GSI PF‐0308414 reportedly demonstrates promising clinical activity in a phase I dose‐finding study.^30^ Some studies have suggested the combined use of low dose GSIs and other drugs to target tumor cells, with the objectives of tolerable toxicity and better clinical responses.^31^ Thus, designing rational combinations of therapeutic agents to inhibit possible compensatory escape mechanisms could be of particular importance. Furthermore, targeting several pathways simultaneously may offer considerable benefits. Notably, CDK5 inhibitors are already in pharmaceutical development owing to the broad protumorigenic role of CDK5.^7^ Therefore, the development of CDK5 and Notch as combinational targets for pancreatic cancer could be undertaken. Our data showed that the combination of roscovitine and DAPT exhibited stronger inhibition on PDAC cell proliferation and migration than the inhibition of CDK5 or Notch function alone (Figure 4A and 4C). It has been demonstrated that CDK5 inhibition can sensitize the neovascular endothelium of burgeoning tumors to allow more effective anti‐angiogenic treatment, such as the inhibition of the Dll4/Notch pathway.^19^ Thus, the combinational inhibition of Notch and CDK5 is a potential strategy for PDAC treatment. This combinational strategy need to be further confirmed in xenograft mouse model.

## CONFLICT OF INTEREST

The authors have no conflict of interest to report.

## Supporting information

Figure S1

Figure S2

Figure S3

## Data Availability

The datasets used and analyzed during the current study are available from the corresponding author on reasonable request.
