# Supplementary figures and images for: CDK5 positively regulates Notch1 signaling in pancreatic cancer cells by phosphorylation
Source: Cancer Med. 2021 May 7;10(11):3689–99. doi: 10.1002/cam4.3916 (PMC8178504; doi:10.1002/cam4.3916)

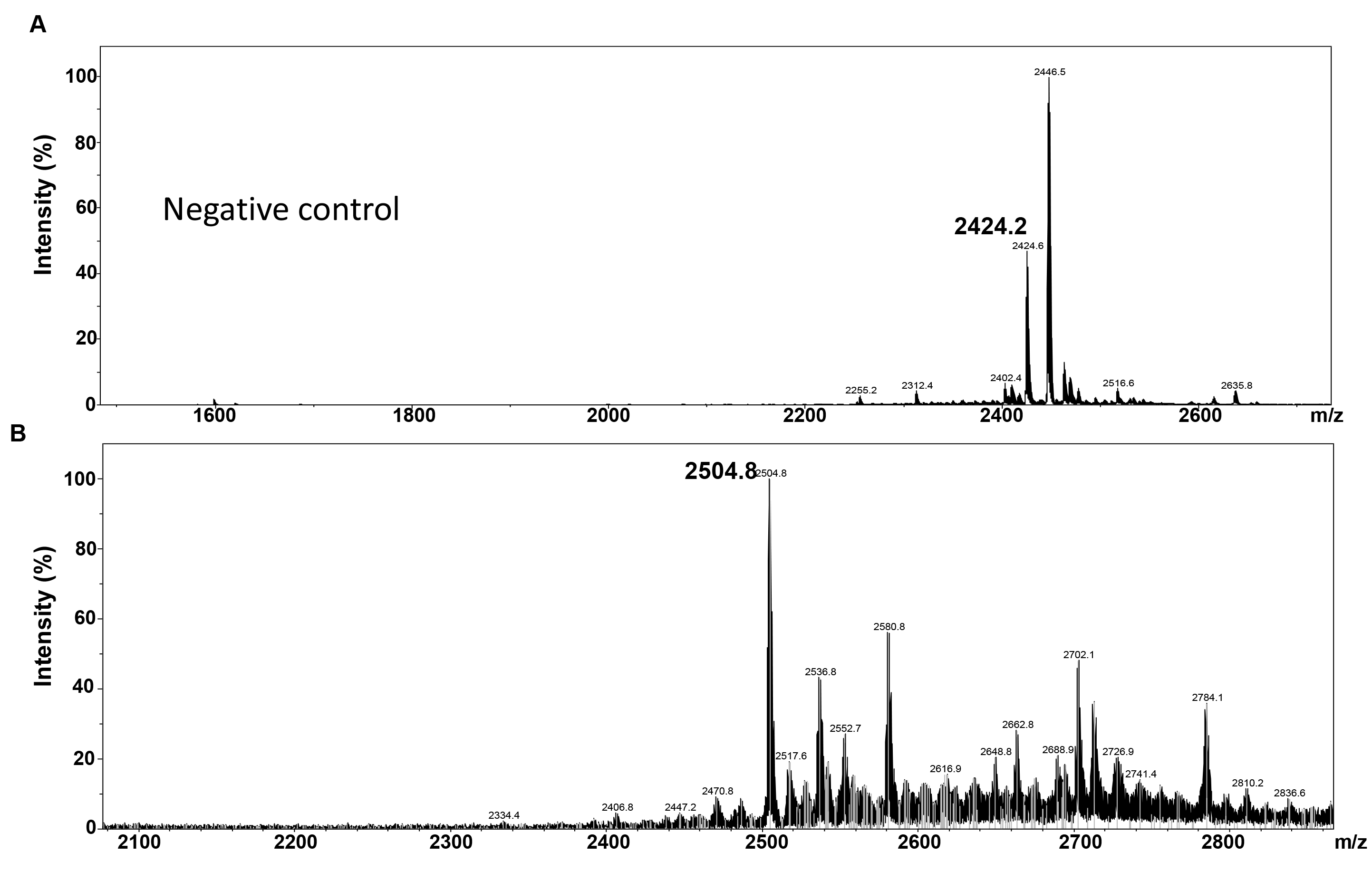

Supplement: Supplementary file 1 — Figure S1 [file CAM4-10-3689-s001.tif]

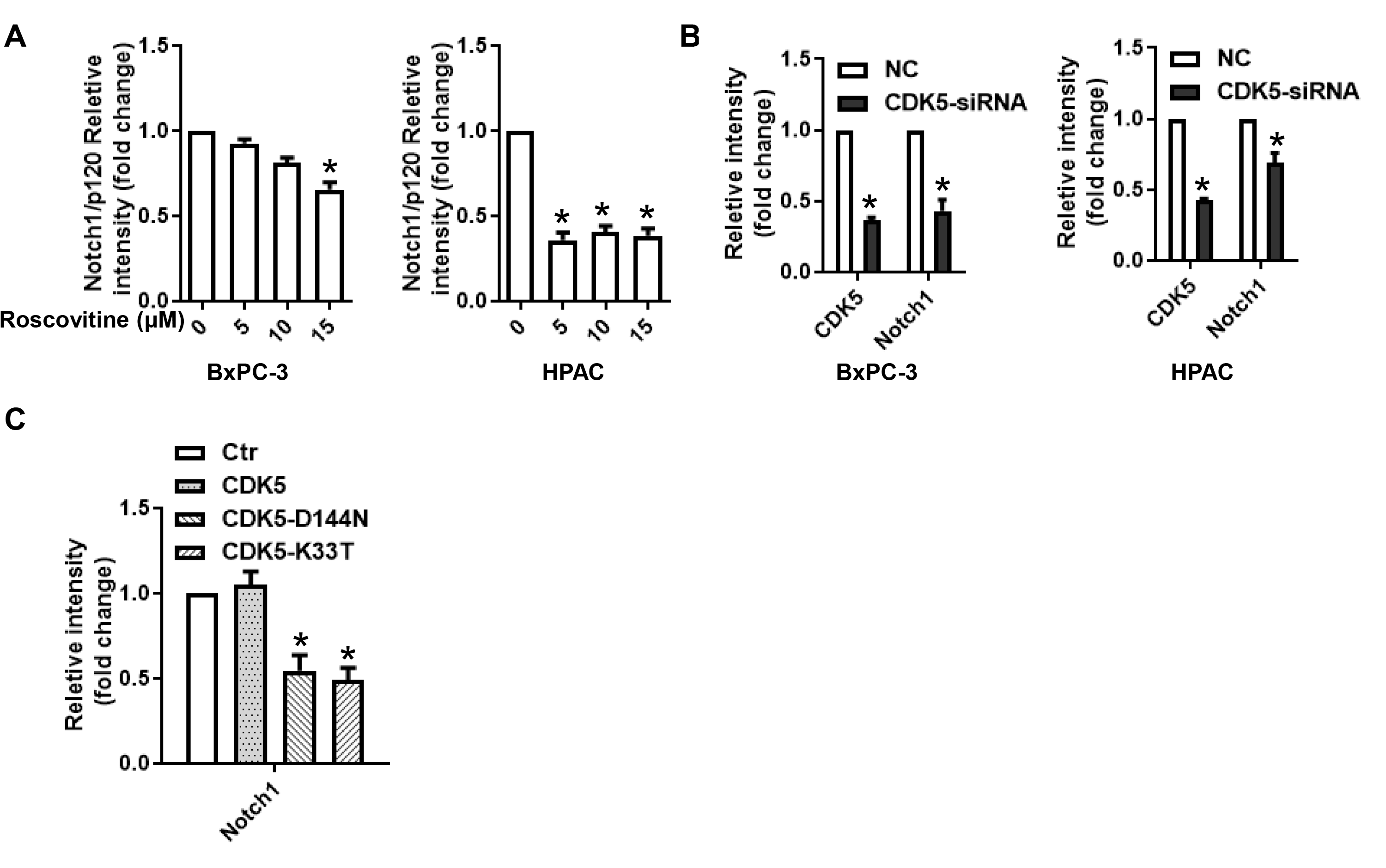

Supplement: Supplementary file 2 — Figure S2 [file CAM4-10-3689-s002.tif]

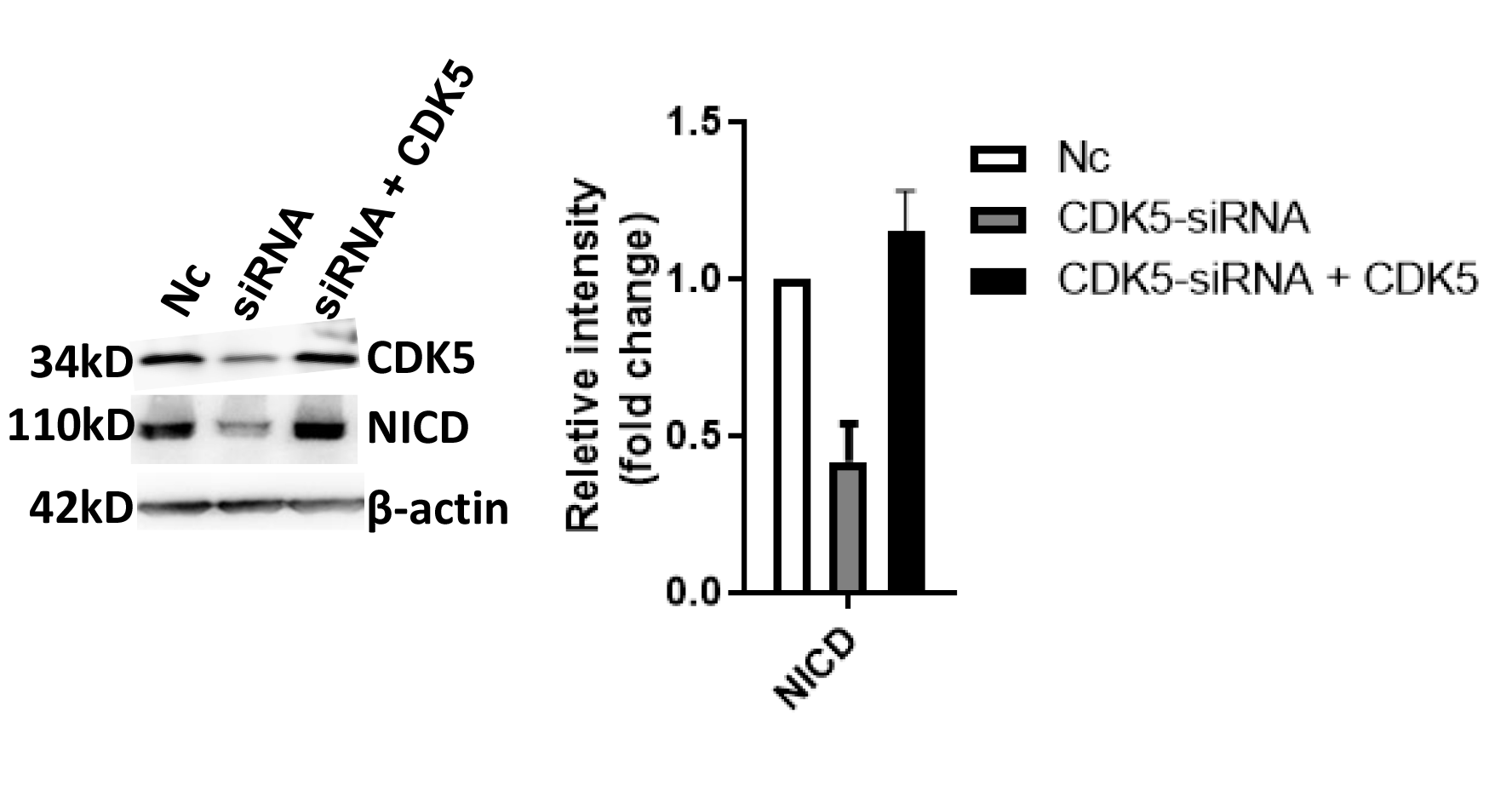

Supplement: Supplementary file 3 — Figure S3 [file CAM4-10-3689-s003.tif]
